# Watchman Device Dislodgement Creating a Left Ventricular Outflow Tract Obstruction Requiring Emergency Cardiopulmonary Bypass

**DOI:** 10.1155/2022/3215334

**Published:** 2022-02-23

**Authors:** Alex Roberts, Steven Mach, Jason Goebel, Heather Palomino, Derek Horstemeyer

**Affiliations:** Grand Strand Regional Medical Center, Myrtle Beach, SC, USA

## Abstract

Left atrial appendage (LAA) occlusion device implantation is becoming a more common alternative for stroke prophylaxis in patients with nonvalvular atrial fibrillation (AF) who are not able to tolerate long-term anticoagulation. Studies suggest the procedure has a 98.5% successful deployment rate (Boersma et al., 2016). We present a case where a rare but known complication involving dislodgement and migration of an implanted Watchman LAA occlusion device led to functional stenosis of the aortic valve creating a left ventricular outflow tract (LVOT) obstruction necessitating emergency cardiopulmonary bypass in the electrophysiology lab to safely retrieve the device.

## 1. Introduction

Implantation of a left atrial appendage (LAA) occlusion device is an increasingly common procedure performed for patients with a history of atrial fibrillation (AF) who have a contraindication to treatment with anticoagulants. These patients are at an increased risk of developing a thrombotic stroke, most commonly originating in the LAA [1, 2]. As of 2015, the Watchman device has been the only left atrial appendage occlusion device available for clinical use in the United States [3]. We present a unique case of a patient presenting for outpatient implantation of a Watchman device complicated by dislodgement, migration, and obstruction of the left ventricular outflow tract (LVOT) leading to cardiopulmonary collapse and requiring emergent cardiopulmonary bypass (CPB) as a life-saving intervention to remove the device.

The patient provided signed informed consent and written Health Insurance Portability and Accountability Act authorization to publish this report. This article adheres to the applicable Enhancing the Quality and Transparency of Health Research (EQUATOR) guideline.

## 2. Case Description

**Figure 1:**
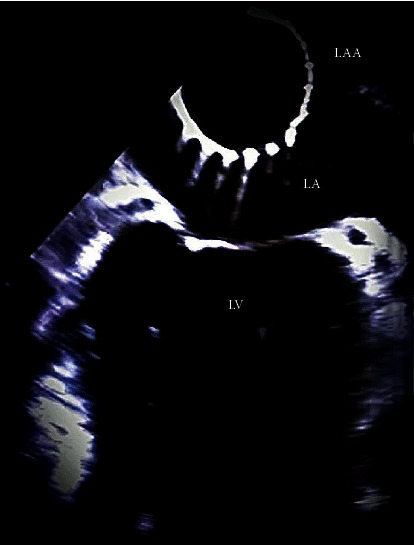
Mid-esophageal 45° TEE view showing the Watchman device floating freely in the left atrium after being dislodged from the left atrial appendage (TEE: transesophageal echocardiogram, LAA: left atrial appendage, LA: left atrium, and LV: left ventricle).

A 79-year-old female with a history of chronic AF on long-term anticoagulation therapy with apixaban 5 mg twice daily, dilated cardiomyopathy, stage 3 chronic kidney disease, and recurrent gastrointestinal bleeding secondary to chronic anticoagulation use presented for elective outpatient Watchman device placement. The patient was hemodynamically stable in AF. Labs were remarkable for a creatinine of 1.5 mg/dL. On physical exam, the patient had an irregular heart rhythm. General endotracheal anesthesia was induced in the electrophysiology suite with midazolam 2 mg, propofol 200 mg, and rocuronium bromide 30 mg. All standard anesthesia monitors were employed, a radial arterial line was placed, and anesthesia was maintained with sevoflurane at 1.0 minimum alveolar concentration. Intraoperative transesophageal echocardiogram (TEE) revealed a baseline ejection fraction (EF) of 35–45%, significant left atrial enlargement, and absence of LAA thrombus. Procedural cannulation of both femoral veins was performed, and systemic intravenous heparin was administered to achieve an adequate activated clotting time prior to trans-septal puncture. A 27 mm Watchman FLX LAA occlusion device was inserted without difficulty, and proper positioning was confirmed using TEE, minimal IV contrast dye, and a tug test showing adequate device recall and opposition to the appendage. Based on meeting the standard position, anchor, size, and seal criteria, the Watchman device was deployed from the delivery system. Shortly after several systolic heartbeats, the device had dislodged from the LAA and was floating freely in the left atrium (Figure 1). Cardiothoracic Surgery (CTS) and Interventional Radiology (IR) were immediately consulted. During a wire-guided attempt to retrieve the device, through a second trans-septal site, the device was noted to have migrated through the mitral valve into the left ventricle. The patient remained hemodynamically stable, and TEE exam revealed good valve motion and blood flow across the mitral valve.

**Figure 2:**
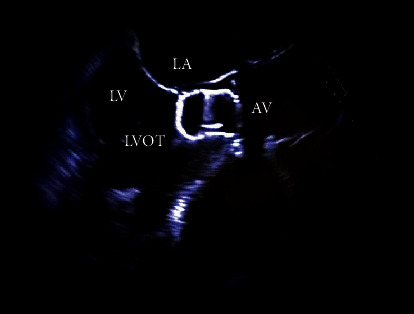
Mid-esophageal aortic valve long-axis TEE view showing the Watchman device occluding the left ventricular outflow tract and aortic valve (TEE: transesophageal echocardiogram, LAA: left atrial appendage, LA: left atrium, LV: left ventricle, LVOT: left ventricular outflow tract, and AV: aortic valve).

Multiple attempts were made to remove the device via the retroaortic approach using a wire to cross the aortic valve to snare the device into the aorta. After several passes, the Watchman device had migrated through the LVOT and lodged anteriorly under the aortic valve leaflets, obstructing the aortic valve (Figure 2). This created a functional aortic stenosis, and the patient's blood pressure dropped to 60/40 mmHg with a heart rate approaching 170 beats per minute. Additional systemic intravenous heparin was given, and the patient was immediately placed on femoral arterial bypass by CTS with a plan to surgically remove the Watchman device once hemodynamically stable. During attempted peripheral cannulation, the patient's rhythm transitioned to ventricular fibrillation (VF) arrest. Advanced cardiac life support was initiated with chest compressions. Norepinephrine 0.1 mcg kg^−1^ min^−1^, vasopressin 0.1 u/min, lidocaine 3 mg kg^−1^ hr^−1^, and dobutamine 5 mcg kg^−1^ min^−1^ infusions were immediately started. A total of eight minutes had elapsed from the time of VF arrest to median sternotomy followed by central cannulation for bypass. Hemodynamic stability was achieved briefly; however, VF recurred and was transcutaneously defibrillated twice without resolution.

Antegrade cardioplegia was used to arrest the heart after cross-clamping the aorta. The proximal aorta was surgically opened, and the Watchman device was removed successfully. The aortotomy was sutured closed, the cross-clamp was removed, and the patient was ventricularly paced to a rate of 80 beats per minute. On repeat TEE exam, the EF was mildly reduced to 30–35%, with both aortic and mitral valves free of any significant damage or deficiencies. The LAA was surgically clipped and deemed appropriate by TEE examination. An initial attempt to separate from CPB was complicated by hypotension and ventricular tachycardia (VT) with recurrence of VF. Multiple defibrillation attempts with internal paddles were unsuccessful, and the rhythm alternated between VT and AF with rapid ventricular response. Amiodarone 150 mg was administered, and CPB was resumed. The patient was slowly weaned off of CPB and was hemodynamically stable without evidence of further arrhythmia. Heparin was reversed with protamine. Packed red blood cells, fresh frozen plasma, platelets, cryoprecipitate, and clotting factor VII were administered to help treat coagulopathy.

The patient was taken to the cardiovascular intensive care unit and intubated with inotropic and vasopressor support. She was extubated several hours later and was neurologically intact, and she alternated between normal sinus rhythm and AF. On postoperative day one, she moved from bed to chair and was eventually able to ambulate with physical therapy around the intensive care unit. She was appreciative and thankful of her care. The patient was discharged on postoperative day 10 to a skilled nursing facility for rehabilitation with plans for clinic follow-up in two weeks and prescribed apixaban 5 mg twice daily for six weeks before transitioning to aspirin 81 mg daily.

## 3. Discussion

The implantation of LAA occlusion devices is becoming more common and is considered to be relatively safe, although complications from dislodgement can lead to obstructive cardiac shock. While there are several case reports of known dislodgement of Watchman devices, to our knowledge, this is the first case occurring immediately after implantation, involving migration and obstruction of the aortic valve and causing life-threatening cardiovascular collapse requiring emergent CPB and aortotomy. It is estimated that the rate of device dislodgement and embolization is 3.9% [4]. Other reports note instances of migration of the device to the left atrium, left ventricle, or abdominal aorta ranging from intraprocedurally and same day postprocedurally to several months after implantation, respectively [5]. Several instances of device dislodgement have been noted as incidental findings on follow-up imaging that resulted in planned, as opposed to emergent, surgery for removal.

Prior studies including the PREVAIL trial have shown Watchman device implantation to be a reasonable and noninferior alternative to warfarin therapy for stroke prevention in patients with nonvalvular AF [6]. However, although the PROTECT AF trial has suggested that there is a significantly higher risk of complications, predominantly pericardial effusion and procedural stroke related to air embolism compared to anticoagulation alone, the functional impact and safety profile favor Watchman device implantation [7]. While minimally invasive techniques may be utilized for device retrieval after dislodgement and migration, the literature has shown surgical removal to be the favored treatment option when these are unsuccessful or technically difficult [5], as was quickly demonstrated in our case.

Although placement of the Watchman device met all standard position, anchor, size, and seal criteria, it is suspected that the challenging patient anatomy played a role in its dislodgement. In order to reach a coaxial angle in the LAA, there was noted to be a significant amount of torque required on the access sheath to hold the device orthogonal to the ostium of the appendage for deployment. It is believed that when the torque was relaxed, some of the anchors of the device may have come loose, leading to dislodgement. The Watchman device was appropriately sized before insertion via standard TEE measurements including LAA length and ostium width at several angles, and it is not believed that a different sizing would have prevented this complication. In order to ensure proper device seating, it is recommended to implant the device as orthogonal as possible with the least amount of torque on the access sheath. While there are case reports available that describe device dislodgement and embolization beyond the aortic valve into the descending abdominal aorta that are largely asymptomatic [5], in this case, further migration into the LVOT presented as life-threatening hemodynamic instability. It is suspected that the device may have entered the aortic valve while the cusps were closing, becoming trapped. Another possibility is that the aortic valve diameter was notably smaller than the mitral valve diameter which could lead to occlusion, although these were not measured at the time of the procedure given the impending arrest and need for emergency surgery. The patient did not have any history of aortic valve disease, such as a bicuspid valve, or any evidence of excessive valve calcification on TEE exam which could have contributed to the device becoming lodged in the LVOT.

We describe the rare case of a Watchman LAA occlusion device dislodgement and migration through the mitral valve and obstruction of the aortic valve and LVOT causing immediate cardiovascular collapse. In addition to advanced cardiac life support resuscitation, the patient was treated with emergency CPB and aortotomy for device removal. In an emergent situation with impending hemodynamic collapse, the swift decision to proceed with CPB for surgical removal resulted in a life-saving outcome.

## Data Availability

This is a case report, and the underlying data can be found in medical records at Grand Strand Regional Medical Center in Myrtle Beach, SC, USA.

## Abbreviations

AF: Atrial fibrillation
LVOT: Left ventricular outflow tract
LAA: Left atrial appendage
CBP: Cardiopulmonary bypass
TEE: Transesophageal echocardiography
EF: Ejection fraction
CTS: Cardiothoracic surgery
IR: Interventional radiology
VF: Ventricular fibrillation
VT: Ventricular tachycardia.
